# Short Physical Performance Battery (SPPB) and Its Relationship with the Predisposition to Muscle and Joint Injuries Associated with the COL1A1 and IL-6 Gene in Older Adults

**DOI:** 10.3390/jfmk9040215

**Published:** 2024-11-01

**Authors:** Katherine González-Ruíz, Maryleysi Ararat-Sandoval, Shirley Camayo-Guevara, Laura Rojas-Salazar, Leidy Tatiana Ordoñez-Mora, Ilem D. Rosero

**Affiliations:** Health and Movement Research Group, Physiotherapy Program, Faculty of Health, Universidad Santiago de Cali, Santiago de Cali 760033, Colombia; katherine.gonzalez07@usc.edu.co (K.G.-R.); marileysi.ararat00@usc.edu.co (M.A.-S.); shirley.camayo00@usc.edu.co (S.C.-G.); laura.rojas07@usc.edu.co (L.R.-S.); ilemdayana@gmail.com (I.D.R.)

**Keywords:** older adult: SPPB, genes, physical activity, COL1A1, IL-6

## Abstract

**Background/Objectives**: Aging leads to physiological changes influenced by lifestyle, environment, and genetics, increasing the risk of morbidity and mortality in older adults. COL1A1 gene encodes an essential protein in connective tissues, which is associated with musculoskeletal lesions. The interleukin-6 (IL-6) gene is a proinflammatory and anti-inflammatory regulator and has a greater predisposition to fractures and osteoporosis reported. In turn, these alterations are associated with a decrease in physical capacity, leading to a progressive loss of functionality and quality of life in older adults. **Methods**: A cross-sectional study was designed to identify the relationship between physical condition as determined using the Short Physical Performance Battery (SPPB) and the predisposition parameters for muscle and joint injuries in a population of 422 older adults, active and of ≥60 years. The variables evaluated were sociodemographic data, SPPB evaluation, and COL1A1 and IL-6 gene DNA extracted by buccal scraping. **Results**: SPPB total score was significantly correlated with age −0.07, weight −0.02, waist circumference −0.02, and body mass index −0.05 (*p* < 0.005). **Conclusions**: Regarding genetic variables, there were no significant differences. However, lower SPPB values were observed in the GG genotype and GT of COL1A1 when compared to the CC genotype and GC of IL-6.

## 1. Introduction

Aging is a natural and gradual process that affects all living beings and involves morphological, physiological, biochemical, psychological, and social changes. These changes can be influenced by different genetic, environmental, and lifestyle factors. One of the fundamental aspects of aging is the gradual decline in the body’s ability to maintain homeostasis, which is the internal balance necessary for optimal functioning. This occurs when the body’s cellular repair systems and adaptive responses become less efficient over time. Some of the most common consequences of aging include increased vulnerability to noncommunicable diseases, frailty, sarcopenia, and decreased physical and functional capacity [1,2].

According to the World Health Organization (WHO), by 2030, it is estimated that one in six people will be 60 or older. At that time, the number of people over 60 will have increased from 1 billion in 2020 to 1.4 billion. Furthermore, it is projected that by 2050, the global population of people over 60 will double to 2.1 billion [3]. In Colombia, the National Administrative Department of Statistics (DANE, Departamento Administrativo Nacional de Estadística in Spanish) estimated in 2018 that by 2020, there would be a total of 6,808,641 people over the age of 60, representing 13.5% of the projected Colombian population [4]. It is expected that in 2023, there will be about 410,216 people over 60 or older in Santiago de Cali. Of this group, approximately 60% are women and 40% are men [5].

In this context, with aging comes a decline in physical performance and physical qualities, such as flexibility, speed, endurance, and muscular strength [6]. These factors have functional implications, such as decreased gait speed, gradual loss of muscle mass, increased risk of falling, and the resulting progressive loss of independence, not only affecting patients’ physical condition but also the quality of their social, economic, and everyday lives. This leads to an increased risk of disability and death in older adults [7].

Regarding the genetic component, some polymorphisms such as COL1A1 Sp1 and COL1A1 rs1800012 are associated with degenerative musculoskeletal diseases, increasing the risk of fracture [8,9]. Also, interleukin-6 (IL-6) gene variants have been described to have both proinflammatory and anti-inflammatory functions. However, IL-6 is known as a primary regulator of acute and chronic inflammatory processes associated with various autoimmune, musculoskeletal, and endocrine diseases, among others [10]. A recent meta-analysis reported that some polymorphisms of the IL-6 174G/C (rs1800795) and 572C/G (rs1800796) genes have been associated with an increased predisposition to fractures and osteoporosis in postmenopausal women [11,12].

To that effect, functional limitations and genetic predisposition to certain musculoskeletal alterations pose a challenge in public health, and the assessment of physical conditions is essential to identify and monitor the clinical evolution of the functional status of the older adult. SPPB has become one of the main tools for assessing the physical function of older adults. It assesses four aspects of mobility: balance, speed, gait, and lower extremity strength. Furthermore, it has been proposed as a reliable tool to characterize frailty status and all-cause mortality risk in older adults [13]. Therefore, the objective of this study was to identify the relationship between SPPB-assessed physical condition and predisposition parameters to muscle and joint injuries in a population of older adults in Cali, Colombia.

## 2. Materials and Methods

### 2.1. Participants

This was a descriptive, quantitative, cross-sectional study. For the sample calculation, a proportion-based formula was used, considering the population size of older adults as 6,808,641 [4], with a 95% confidence level and a 5% margin of error, resulting in a sample size of 384 participants. An additional 10% adjustment for potential losses brought the total sample size to 422 active individuals aged over 60 years—both men and women, non-institutionalized—recruited through the “Mas Vitales” program of the Secretary of Sports of Santiago de Cali, using non-probabilistic convenience sampling.

This program operates in four zones (including the city’s communes) and districts of the Special District of Santiago de Cali, serving 6254 beneficiaries. For this study, we focused on Zone 3, specifically in communes 17, 19, and 20, which collectively have 800 beneficiaries. Zone 3 was selected for its high sociodemographic variability to generate a representative sample of the target population.

We excluded older adults with moderate to severe cognitive impairment (Minimental scores below 24 points) [14], uncontrolled cardiovascular diseases, and individuals with neurological disorders (confirmed by a recent medical report), acute trauma, visual or hearing impairments, or any health conditions preventing them from participating in physical exercise interventions.

Data were collected at a single time point over 12 months, from August 2022 to July 2023. There were no losses or participants who declined to participate. To minimize potential errors and assessment bias, a standard operating procedure manual was developed, detailing all the assessments to be conducted. As a quality control measure, the primary data were periodically reviewed to detect inconsistencies or missing entries in the database.

### 2.2. Anthropometric Characteristics

Height (m) and weight (kg) were measured. Participants were asked to remove their socks and shoes and stand in the center of the SECA 217 stadiometer with their head erect, gaze straight ahead, feet slightly apart at 60°, and heels, buttocks, shoulders, and the back of the head in contact with the stadiometer. They were instructed to breathe deeply at the time of measurement. For the weight measurement, participants stepped onto the GMD scale wearing as little clothing as possible. A user profile was created, recording sex, age, height, and level of physical activity, which provided results including weight, fat mass percentage, muscle mass, and body mass index (BMI).

Finally, waist circumference was measured. Participants lifted their clothing to expose the waist, and the tape measure was placed at the level of the mid-axillary line. The measurement was taken at the midpoint between the costal ridge and the iliac crest and recorded in centimeters. All anthropometric measurements were taken in the morning, following the International Standards for Anthropometric Assessment (ISAK) guidelines [15]. Additionally, all measurements were conducted by trained research assistants to minimize coefficients of variation.

### 2.3. Short Physical Performance Battery (SPPB)

The SPPB evaluates three categories of mobility in older adults, subdivided into the following: static balance, walking 4 m, and lower limb strength (chair stand test). Participants could obtain a score of 1 to 4 in each of the three sections assessed; then, the three sections are added to obtain the overall score, resulting in 0 (worst) to 12 points (best). A total score of less than 10 indicates frailty and a high risk of disability and falling.

### 2.4. DNA Extraction and Sampling Quantification

The Quinque et al. protocol was used to extract the DNA sample from buccal cells and store them in the Milli-Q water system. Each sample was coded and assigned a consecutive number. For DNA extraction, 2 mL of the buccal scraping mixture and Milli-Q water were taken, to which 30 µL of proteinase K and 150 µL of 10% sodium dodecyl sulfate were added, followed by 400 µL of 5 M sodium chloride. These were then incubated on ice for 10 min. The extracted samples were quantified using a Thermo Scientific Nano Drop 2000/2000c (Waltham, MA, USA) spectrophotometer. Genotyping was performed for each locus through amplification using the polymerase chain reaction from DNA located on autosomal chromosomes with primers for COL1A1 and IL-6 gene polymorphisms. The restriction fragment length polymorphism technique was used to determine the genotype of the genes to be studied, followed by a final elongation of 72 °C, for 10 min [9].

### 2.5. Statistical Analysis

A descriptive analysis was performed for the sociodemographic and SPPB variables. Quantitative variables were described by mean (x) and standard deviation (SD) values, while qualitative variables were described by absolute frequency (n) and percentage frequency (%) values. To estimate the association of the SPPB variable with the gene and sociodemographic variables, a generalized linear model was used (family: normal; link function: identity). To evaluate the effect of genotype, a dominant and recessive allele was used, and it was determined that, without predisposition, muscle and joint lesions in IL-6 and COL1A1 genes, respectively, correspond to the homozygous genotype of allele G. All variables were analyzed using the Statistical Package for Social Sciences version 25, with a statistical significance level value of *p* < 0.05 as reference.

### 2.6. Ethical Considerations

The research study was approved by the Scientific Committee of Ethics and Bioethics of the Universidad Santiago de Cali, Faculty of Health, in the session of 5 November 2021, under Record No. 18. All participants were informed in person about the purpose of the study as well as the benefits and risks. All participants signed their informed consent [16]. The study design guaranteed patients’ privacy and complied with the guidelines established in the Declaration of Helsinki [17].

## 3. Results

### 3.1. Descriptive Characteristics

The sample consisted of 422 older adults: 29 men and 393 women. Table 1 shows participants’ sociodemographic variables and body composition. The mean age of the sample was 72 ± 6.92 years; height was 1.52 ± 7.50 cm; weight was 62.40 ± 10.96 kg; BMI was 27.25 ± 4.34; and waist circumference was 88 ± 10.38 cm. In total, 84.2% of the older adults had an educational level less than or equal to high school, and 45.7% belonged to stratum 1.

Regarding the genetic variables in the population studied, the frequency of the IL-6 GC genotype was 47.9%, and the frequency of the COL1A1 GT genotype was 30.8%.

Table 2 presents the evaluation of physical condition using SPPB. From this, it was evident that 97.5% of the population presented a slight or minimal limitation in functional capacity. Regarding the total SPPB score, women obtained an average of 10.47 ± 1.52 and men 10.34 ± 1.49 for a total population average of 10.46 ± 1.52.

### 3.2. Correlation Between Physical Condition and Sociodemographic, Anthropometric, and Genetic Variables

Table 3 describes the correlation of SPPB with sociodemographic, anthropometric and genetic variables. The SPPB total score was significantly correlated with age −0.07 (−0.09 to −0.05), weight −0.02 (−0.03 to −0.01), waist circumference −0.02 (−0.03 to −0.01) and BMI −0.05 (−0.08 to −0.01) (*p* < 0.005). There were no significant differences in the variables sex, educational level, and socioeconomic stratum.

There were no significant differences regarding genetic variables. However, lower SPPB values were observed in the GG genotype −0.36 (−0.83 to 0.12) and GT of COL1A1 −0.06 (−0.37 to 0.25) when compared to the CC genotype 0.38 (−0.09 to 0.84) and GC of IL−6 0.01 (−0.28 to 0.30).

Although the models exhibit low goodness of fit, with an *R*^2^ of 0.119 and an adjusted *R*^2^ of 0.103, some variables show statistical significance. This suggests that, while the model as a whole does not explain much of the variability in SPPB scores, certain individual variables may still play an important role in predicting performance. These findings emphasize the need to further explore the impact of sociodemographic, anthropometric, and genetic factors on physical function. Additionally, future research should consider incorporating other variables that could enhance the predictive power of the model.

When analyzing the Pearson correlations between the SPPB total score and other variables (Age, BMI, and Waist circumference), the following relationships were observed:

Age: This is the only variable showing a significant moderate negative correlation with SPPB (r = −0.318, *p* < 0.01), indicating that as age increases, the SPPB score tends to decrease. This aligns with theoretical expectations, as physical performance, assessed by the SPPB, generally declines with age.

BMI: A weak negative correlation is observed between BMI and SPPB (r = −0.101, *p* < 0.05), suggesting that higher BMI is slightly associated with a lower SPPB score. Although statistically significant, the strength of the relationship is minimal.

Waist circumference: This variable also demonstrates a weak negative correlation with SPPB (r = −0.124, *p* < 0.05). Like BMI, its association with SPPB is statistically significant but limited in magnitude.

## 4. Discussion

Due to the current increase in life expectancy and decrease in birth rate, there has been an increase in the population of older adults [18]. As we age, systemic changes occur, leading to a gradual decrease in functional, cognitive, and social capacity. This, in turn, is related to an increased susceptibility to noncommunicable and musculoskeletal diseases [7].

Study results related to physical function show that only 2.5% of the population had a severe (0–4) or moderate (4–6) limitation in the SPPB total score, indicating a low risk of frailty and physical performance (score ≥3 and ≤9) as proposed by the European Working Group on sarcopenia in Older People (EWGSO2) [7]. Likewise, a study conducted in a group of 197 older adults who took part in a physical activity program in the city of Cali, Colombia, in 2019 showed that 87.3% of the study population was minimally or mildly limited [19]. This trend coincides with a study conducted in the Basque Country (Spain) in which it was shown that older adults who belong to a program that promotes healthy habits have lesser frailty compared to the general population [20]. It should be noted that our study population also takes part in a program that promotes physical activity, and nearly 40% meet the minimum physical activity recommendations proposed by WHO. It has been shown that higher levels of physical activity and lower levels of sedentary behaviors are related to a better overall score on the SPPB [21,22].

Contrarily, our findings showed that variables associated with cardiovascular risk, such as weight, BMI, and waist circumference, had an inversely proportional relationship with SPPB (*p* < 0.05). These findings are in agreement with those reported by Belletiere B et al. [23], who monitored 5043 older adult women for six years and found that a lower SPPB score was associated with a higher risk of cardiovascular disease. Similarly, Pavasini P et al. [24] performed a meta-analysis and determined that an SPPB score of less than 10 (0–3, 4–6, and 7–9) was associated with an increased risk of all-cause mortality (odds ratio [OR] = 3.35, 2.14, and 1.50, respectively). It has been described that the loss of muscle strength increases the risk of developing cardiovascular disease as a result of increased oxidative stress, inflammation, and central adiposity characteristic of old age [25]. In this sense, objective measurements such as SPPB through components such as gait speed, muscle strength, and functionality can be considered prognostic indicators of health in older adults [24].

Similarly, in our study, we found an inversely proportional association between age and total SPPB score. It has been described that as age increases, there is a gradual decrease in muscle strength, affecting mainly the antigravity muscles due to neurophysiological changes, predominant atrophy of type I fibers, and decrease in collagen, leading to an increase in the grade of tendon stiffness, a modification of joint biomechanics and an increased risk of connective tissue and musculoskeletal injury [26]. Likewise, Quintero et al. [27] demonstrated that after 75 years, there is an annual loss of 3.4% of muscle strength due to a decrease in functionality, an increase in physical dependence, and, in turn, a lower quality of life in older adults. Nevertheless, a large body of evidence supports that regular physical exercise is associated with a lower risk of noncommunicable diseases and a higher grade of independence and well-being in older adults [28,29,30].

In addition, genetics also plays an important role in the development and understanding of the aging process. Currently, different investigations have described some genes and polymorphisms as causal agents for the onset of some diseases associated with physical, musculoskeletal, and cognitive function in older adults [31,32,33]. For example, our study identified a frequency of 47% of the GC IL-6 genotype and 30.8% of the GT genotype of COL1A1, in which both contained a heterozygous genotype, indicating that the population has a medium predisposition to musculoskeletal injuries, such as fractures, osteoporosis, and loss of muscle strength. In this sense, Wang, C et al. [33] reported that the COL1A1 Sp1 rs1800012 T/T polymorphism is associated with various connective tissue disorders affecting the properties of type I collagen, which causes a greater risk of injuries such as osteoarthritis, osteoporosis, and soft tissue injuries such as rupture of the anterior cruciate ligament [33,34]. These results are similar to those described by Zhong et al. [8], who identified that the COL1A1 gene with G/T polymorphism alters the DNA–protein interaction, increasing the amount of α1 chains of type I collagen. This leads to the alteration of the ratio between α1 and α2 chains (2:1), increasing the binding of RNA polymerase II, which contributes to a greater predisposition to musculoskeletal lesions.

As for the IL-6 gene, in a study conducted in China, Yang et al. [35] demonstrated that −572C/G and −174G/C polymorphisms of the IL-6 gene reduce muscle performance due to the loss of muscle mass. This, in turn, relates to a gradual loss of functionality and a greater risk of suffering cardiovascular and metabolic diseases. According to the study conducted by Castro et al. [36], the rs18000795 polymorphism of the IL-6 gene is related to cardiovascular diseases. In addition, he emphasized that genetic susceptibility does not always condition the same alteration in all populations since it can vary between different population groups and generate a different predisposition.

In this context, it should be noted that no correlation was found between the polymorphisms of the heterozygous GC allele of the IL-6 gene and the heterozygous GT allele of the COL1A1 gene and the total SPPB score. However, although there were no statistically significant differences, lower SPPB values were observed in the homozygous G genotype and heterozygous GT genotype of the COL1A1 gene when compared with the homozygous C genotype and heterozygous GC genotype of the IL-6 gene.

These results are related to those reported by Custodero et al. [37], who evaluated 289 older adults and found that a state of low-grade chronic inflammation (high IL-6 levels) was associated with a decrease in gait speed, lower physical performance in lower limbs and low strength, evaluated with SPPB. Conversely, Rubio et al. [38] found a significant correlation between the total SPPB score and the total weekly METs intake; that is, higher levels of physical activity were related to a better SPPB score and a lower rate of muscle mass loss, leading to better physical qualities and functionality in general [39].

In this context, maintaining physical function and independence in older adults becomes a challenge for governmental entities worldwide [40] since it requires the implementation of public policies and programs aimed at promoting active aging, which guarantee the inclusion of the entire community without distinction of origin or socioeconomic stratum.

Regarding the control of potential bias in the study: To mitigate selection bias, random sampling was employed, or clear inclusion criteria were defined to ensure participants were representative of the target population. Recall bias was minimized by reducing reliance on self-reported information, prioritizing objective physical performance data, and conducting assessments in real time to lower the chance of recall errors. Finally, information bias was addressed by standardizing the SPPB scale application procedure, training assessors, and using validated tools to prevent systematic errors in data collection.

Limitations: At present, there were no national or Latin American benchmarks to be found, so the research is based on the cut-off points from the study conducted in the Basque Country (Spain) for the SPPB score: severe (0–4 points), moderate (4–6 points), mild (7–9 points), and minimal (10–12 points) limitation [20].

## 5. Conclusions

The aging process entails significant changes in systemic health that impact on individuals’ functional capacity, increasing vulnerability to noncommunicable and musculoskeletal diseases. In addition, a clear association has been observed between cardiovascular risk factors and functionality, indicating that lower exposure to these factors is related to a more gradual decline in functional capacity over time.

Although in our study, the IL-6 GC and COL1A1 GT genetic polymorphisms were not found to be directly related to the SPPB total score, it highlights the importance of investigating genetics in relation to functionality, as there is previous evidence suggesting inflammation plays a role in decreased functionality, particularly for high IL-6 levels.

It is crucial to recognize that genetics is only one of multiple factors that influence functionality and that outcomes may vary in different populations and contexts. This study highlights the need to continue to explore the complex interactions between genetic, environmental, and lifestyle factors that influence functionality in old age.

## Figures and Tables

**Table 1:** Sociodemographic characteristics, body composition, and genes.

Variables	Woman *n* = 393	Man *n* = 29	Total *n* = 422
Short Physical Performance Battery _x(s)_	10.47 (1.52)	10.34 (1.49)	10.46 (1.52)
Size _x(SD)_	150.45 (6.66)	162.84 (8.70)	151.30 (7.50)
Weight _x(SD)_	61.97 (10.85)	68.21 (11.03)	62.40 (10.96)
Body mass index _x(SD)_	27.35 (4.32)	25.82 (4.39)	27.25 (4.34)
Waist circumference _x(SD)_	87.67 (10.37)	92.53 (9.67)	88.00 (10.38)
**Age group**			
60–64 years old	52 (13.2%)	4 (13.8%)	56 (13.3%)
65–69 years old	100 (25.4%)	3 (10.3%)	103 (24.4%)
70–74 years old	94 (23.9%)	7 (24.1%)	101 (23.9%)
75–79 years old	84 (21.4%)	6 (20.7%)	90 (21.3%)
≥80 years old	63 (16.0%)	9 (31.0%)	72 (17.1%)
**Educational level _n(%)_**			
Illiterate	20 (5.1%)	2 (6.9%)	22 (5.2%)
Incomplete elementary school	98 (24.9%)	9 (31.0%)	107 (25.4%)
Completed elementary school	98 (24.9%)	4 (13.8%)	102 (24.2%)
Completed high school	115 (29.3%)	9 (31.0%)	124 (29.4%)
Undergraduate	52 (13.2%)	4 (13.8%)	56 (13.3%)
Postgraduate	10 (2.5%)	1 (3.4%)	11 (2.6%)
**Social stratum _n(%)_**			
Stratum 1	179 (45.5%)	14 (48.3%)	193 (45.7%)
Stratum 3	94 (23.9%)	6 (20.7%)	100 (23.7%)
Stratum 4	110 (28.0%)	8 (27.6%)	118 (28.0%)
Stratum 5	10 (2.5%)	1 (3.4%)	11 (2.6%)
**IL-6**			
GG	159 (40.5%)	14 (48.3%)	173 (41.0%)
GC	189 (48.1%)	13 (44.8%)	202 (47.9%)
CC	45 (11.5%)	2 (6.9%)	47 (11.1%)
**COL1A1**			
GG	236 (60.1%)	14 (48.3%)	250 (59.2%)
GT	116 (29.5%)	14 (48.3%)	130 (30.8%)
TT	41 (10.4%)	1 (3.4%)	42 (10.0%)

Self-designed study. x, mean; SD, standard deviation; n, absolute frequency; %, percentage frequency; IL-6, Interleukin 6; COL1A1, Collagen type 1 alpha 1; G, Guanine; C, Cytosine; T, Thymine.

**Table 2:** Physical condition (SPPB scores).

Score	Population
Severe (0–4)	3
Moderate (4–6)	5
Mild (7–9)	91
Minimal (10–12)	323

Self-designed study.

**Table 3:** SPPB correlation to sociodemographic, anthropometric, and genetic variables.

Variables	Simple Model		Adjusted Model	
	β (95%CI)	*p*-Value	β (95%CI)	*p*-Value
Size x(s)	0.00 (−0.02 a 0.02)	0.868	−0.01 (−0.03 a 0.01)	0.261
Weight x(s)	−0.01 (−0.03 a 0.00)	0.062	−0.02 (−0.03 a −0.01)	0.001 *
Body mass index x(s)	−0.04 (−0.07 a 0.00)	0.038	−0.05 (−0.08 a −0.01)	0.004 *
Waist circumference x(s)	−0,02 (−0,03 a 0.00)	0.011	−0.02 (−0.03 a −0.01)	0.006 *
Age	−0.07 (−0.09 a −0.05)	0.000	−0.07 (−0.09 a −0.05)	0.000 *
Sex	(a)		(a)	
Woman	0.12 (−0.45 a 0.70)	0.674	−0.04 (−0.59 a 0.51)	0.890
Man	Ref. (a)		Ref. (a)	
Origin	(a)		(a)	
Rural	−0.02 (−0.36 a 0.31)	0.893	0.09 (−0.23 a 0.41)	0.583
Urban	Ref. (a)		Ref. (a)	
Educational level n(%)	(a)		(a)	
Illiterate	0.15 (−0.55 a 0.85)	0.677	0.19 (−0.48 a 0.85)	0.578
Incomplete elementary school	Ref. (a)		(a)	
Completed elementary school	0.39 (−0.02 a 0.81)	0.063	0.28 (−0.11 a 0.68)	0.160
Completed high school	0.33 (−0.06 a 0.73)	0.097	0.22 (−0.16 a 0.59)	0.256
Undergraduate	0.27 (−0.23 a 0.76)	0.288	0.03 (−0.44 a 0.51)	0.890
Postgraduate	0.33 (−0.62 a 1.28)	0.493	−0.18 (−1.09 a 0.73)	0.698
Social stratum n(%)	(a)		(a)	
Stratum 1	Ref. (a)		(a)	
Stratum 3	0.27 (−0.10 a 0.64)	0.154	0.17 (−0.18 a 0.52)	0.339
Stratum 4	0.13 (−0.22 a 0.48)	0.463	0.20 (−0.13 a 0.54)	0.232
Stratum 5	0.28 (−0.64 a 1.21)	0.547	0.09 (−0.79 a 0.98)	0.835
IL-6	(a)		(a)	
GG	Ref. (a)		(a)	
GC	0.01 (−0.30 a 0.32)	0.947	0.01 (−0.28 a 0.30)	0.945
CC	0.40 (−0.09 a 0.89)	0.112	0.38 (−0.09 a 0.84)	0.113
COL1A1	(a)		(a)	
GG	Ref. (a)		(a)	
GT	−0.04 (−0.36 a 0.28)	0.815	−0.06 (−0.37 a 0.25)	0.690
TT	−0.29 (−0.78 a 0.21)	0.261	−0.36 (−0.83 a 0.12)	0.141

Self-designed study. β: beta coefficient; 95% CI: 95% confidence interval. Adjusted Model: age and sex. * *p* < 0.05. Ref. Reference. IL-6, Interleukin 6; COL1A1, Collagen type 1 alpha 1; G, Guanine; C, Cytosine; T, Thymine. a: reference category.

## Data Availability

The data are not publicly available due to privacy.
